# Privacy-Preserving Prediction of Postoperative Mortality in Multi-Institutional Data: Development and Usability Study

**DOI:** 10.2196/56893

**Published:** 2024-07-05

**Authors:** Jungyo Suh, Garam Lee, Jung Woo Kim, Junbum Shin, Yi-Jun Kim, Sang-Wook Lee, Sulgi Kim

**Affiliations:** 1 Department of Urology Asan Medical Center, University of Ulsan College of Medicine Seoul Republic of Korea; 2 CryptoLab Inc Seoul Republic of Korea; 3 Department of Environmental Medicine Ewha Womans University College of Medicine Seoul Republic of Korea; 4 Department of Anesthesiology and Pain Medicine Asan Medical Center, University of Ulsan College of Medicine Seoul Republic of Korea

**Keywords:** machine learning, privacy, in-hospital mortality, homomorphic encryption, multi-institutional system

## Abstract

**Background:**

To circumvent regulatory barriers that limit medical data exchange due to personal information security concerns, we use homomorphic encryption (HE) technology, enabling computation on encrypted data and enhancing privacy.

**Objective:**

This study explores whether using HE to integrate encrypted multi-institutional data enhances predictive power in research, focusing on the integration feasibility across institutions and determining the optimal size of hospital data sets for improved prediction models.

**Methods:**

We used data from 341,007 individuals aged 18 years and older who underwent noncardiac surgeries across 3 medical institutions. The study focused on predicting in-hospital mortality within 30 days postoperatively, using secure logistic regression based on HE as the prediction model. We compared the predictive performance of this model using plaintext data from a single institution against a model using encrypted data from multiple institutions.

**Results:**

The predictive model using encrypted data from all 3 institutions exhibited the best performance based on area under the receiver operating characteristic curve (0.941); the model combining Asan Medical Center (AMC) and Seoul National University Hospital (SNUH) data exhibited the best predictive performance based on area under the precision-recall curve (0.132). Both Ewha Womans University Medical Center and SNUH demonstrated improvement in predictive power for their own institutions upon their respective data’s addition to the AMC data.

**Conclusions:**

Prediction models using multi-institutional data sets processed with HE outperformed those using single-institution data sets, especially when our model adaptation approach was applied, which was further validated on a smaller host hospital with a limited data set.

## Introduction

The demand for combining widespread data from various hospitals to create a larger data set for research from medical researchers is ongoing. However, exchanging or sharing medical data among hospitals is highly challenging because of various regulations and restrictions [1]. Sharing medical data with other institutions is limited owing to concerns over personal data breach. In other words, most medical data are exclusively accessible to each institution, but data access is mutually exclusive, blocking access from other institutions. Owing to strict data protection policies and privacy regulations, various legal and regulatory barriers to transferring patient-level heterogeneous data among institutions exist. However, as predictive studies using large data have been actively conducted in precision medicine recently, demands to compile multi-institutional data and develop widely applicable models in more diverse clinical environments are increasing. Efforts have been invested to address these challenges using emerging privacy-enhancing technologies (PETs), including homomorphic encryption (HE)—a form of encryption that permits calculations directly on encrypted data, ensuring the security of both input and output of a numerical model [2-5]. It has been demonstrated to be effective in specific “privacy-preserving data sharing and analytics” contexts, for tasks such as delegated computation (wherein data are processed by a third party without revealing its content) or generating summary statistics without exposing individual raw data [6,7]. However, owing to HE’s inherent computational constraints, several HE applications have primarily focused on computationally simpler tasks, such as computing summary statistics. Nevertheless, recent advancements in HE technology have evolved to a stage wherein the development or training of predictive models—particularly with large data sets in multi-institutional studies—has become achievable.

Recent advancements in privacy-preserving techniques in medical data analysis have significantly influenced the field, particularly through the use of HE. Several studies have explored the application of HE for privacy-preserving logistic regression and collaborative learning. For example, Kim et al [8] demonstrated the feasibility of training logistic regression models on homomorphically encrypted data, while Bos et al [9] applied HE to enable secure genome-wide association studies. Furthermore, the scalability of HE-based logistic regression has been demonstrated by Crawford et al [10], who successfully trained 30,000 models on encrypted data.

Our study distinguishes itself from previous works by applying HE to enable secure multi-institutional learning for postoperative mortality prediction using real-world clinical data. In addition, we propose a method called “model adaptation” strategy that allows smaller institutions to leverage encrypted data from larger institutions, improving their predictive models’ performance without compromising patient privacy. By focusing on developing a predictive model through multi-institutional collaboration and emphasizing the practical applicability of our approach, our study pushes the boundaries of privacy-preserving medical data analysis and offers tangible solutions for enhancing predictive modeling in a secure, collaborative manner.

This study, aiming to verify the feasibility of securely developing a predictive model with multi-institutional data sets, focused on protecting each institution’s data set using HE technology. Furthermore, we sought to determine whether the predictive performance can be improved by merging various multi-institutional data sets to project the 30-day postoperative mortality rate. Additionally, we showcased the application of our proposed “model adaptation” strategy. By supplementing and learning from a small amount of data based on an HE-encrypted large-scale data set from external institutions, institutions can construct a predictive model applicable within their clinical setting.

## Methods

### Ethical Considerations

This study was approved by the Institutional Review Board (IRB) of the Asan Medical Center (AMC) (IRB No. 2021-0186) and Ewha Womans University Medical Center (EUMC) (IRB No. 2020-11-017). The requirement of obtaining written informed consent was waived owing to the retrospective study design. We used the publicly available INSPIRE data set provided by the Seoul National University Hospital (SNUH). The composition, release, and usage of the INSPIRE data set were separately approved by the SNUH’s IRB (H-2210-078-1368).

### Inclusion and Exclusion Criteria

We retrospectively analyzed data collected from 341,007 patients aged 18 years and older who underwent noncardiac surgeries at 3 independent institutions. The data collection period for SNUH patients who underwent noncardiac surgeries was adjusted to January 2011 to December 2020, resulting in the inclusion of 46,956 patients. Moreover, we obtained data from 162,184 patients who underwent surgeries between January 2017 and April 2021 at the AMC. The apparent disparity in the number of patients between these institutions primarily stems from the mapping of our data set with the pre-existing public database, VitalDB. Additionally, our data set included 131,867 patients who underwent surgeries between January 2001 and December 2019 at the EUMC. Patients who had undergone cardiac procedures, organ transplantations, and neurosurgical operations, as well as those with an indeterminable final clinical outcome because of insufficient follow-up within the study timeframe, were excluded. Our analysis only incorporated the first surgical procedure post-admission for patients who had undergone several surgeries within the study period.

### Data Collection and Variable Selection

Data encompassing patient demographics, preoperative laboratory evaluations, details of the surgery, and postoperative clinical outcomes were culled from the electronic medical record system of each respective hospital. Variables for the model were selected in the same manner as in the previous study [11]. Features that consistently ranked high across all hospitals were considered potential candidates for subsequent analyses. The study disregarded variables exhibiting substantial disparities among hospitals, a high incidence of missing values, and susceptibility to subjective measurement and execution by medical personnel. Through these processes, the following 19 variables that served as features for our investigation were selected: (1) demographic data (age, sex, and BMI); (2) preoperative laboratory results (white blood cells, hemoglobin, platelets, sodium, potassium, blood urea nitrogen, creatinine, albumin, aspartate transaminase, alanine transaminase, glucose, prothrombin time, and activated partial thromboplastin clotting time); (3) surgery type (general, otolaryngological, urological, orthopedic, gynecological, and plastic); (4) anesthesia type (general, neuroaxial, monitored anesthesia care, and regional); and (5) status of emergency surgery.

During the modeling process with encrypted data, we strictly adhered to the principle of complete ignorance of the data’s content. This approach, integral to our study design, is not merely a precaution; rather, it is essential for ensuring our analysis’ objectivity and reliability. By consciously avoiding any knowledge of the data’s nature, we aimed to minimize potential biases from prior data set familiarity, thus bolstering our findings’ integrity and validity, particularly in encrypted data scenarios.

### Data Preprocessing

All continuous variables underwent scaling using the StandardScaler function from the scikit-learn library, while categorical variables were incorporated into the model using one-hot encoding. We assumed that standardization for each feature had been implemented at every hospital before encryption, using their respective means and standard deviations.

### Model Outcomes

The primary outcome of interest in the study was in-hospital mortality within 30 days postoperatively. Data on in-hospital mortality were procured as binary information, derived from the final mortality date in the electronic medical record within 30 days postoperatively (“1” representing mortality and “0” indicating survival, with a threshold of 0.5).

### Model Building

**Figure 1:**
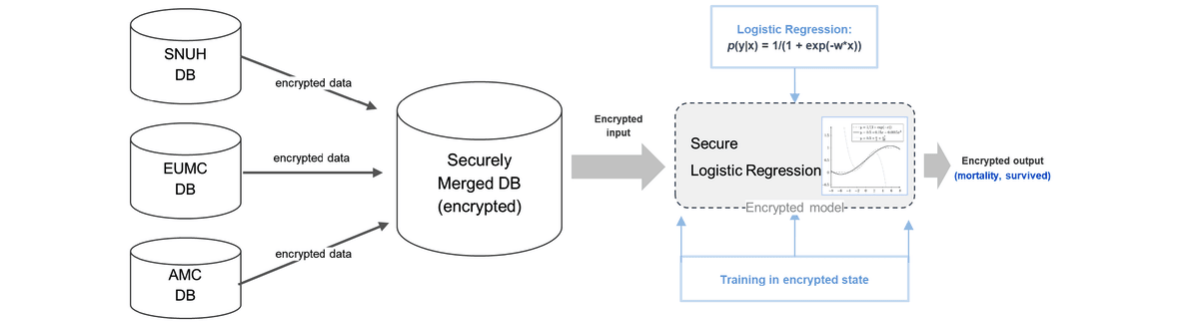
Schematic diagram of external validation of each hospital model. AMC: Asan Medical Center; EUMC: Ewha Womans University Medical Center; DB: database; SNUH: Seoul National University Hospital.

For modeling, secure logistic regression was used to compare the models’ predictive performance (Figure 1) [8]. Complete data sets of each hospital were partitioned into training, validation, and testing data sets using a 6:2:2 distribution to develop all the models. The Nesterov Accelerated Gradient optimizer was applied to build trained models with a learning rate of 0.01 and batch size of 64 [12]. Binary cross-entropy served as the loss function for the model [13], with parameters being optimized to reduce each model’s loss of function to the minimum. To address the imbalance in clinical data and more robustly assess the generalized performance of each model, we use the bootstrap sampling technique [14], which involved repeated processes of resampling training data, creating a new model, and evaluating that model. Thereafter, the model’s performance was quantified as the mean performance of separate models developed with the bootstrap approach. Overfitting issues can be mitigated by averaging their results, thus increasing the model’s generalizability. Consequently, the bootstrap method can significantly diminish the developed models’ overfitting. To validate the predictive performance, the model was evaluated using the test set of the chosen hospital and data gathered from the amalgamations of other hospitals. For a fair comparison, we used the plaintext version of logistic regression for unencrypted data using NumPy from scratch [15], based on a stochastic approach, as opposed to using scikit-learn.

### Model Adaptation

We proposed a methodology for “model adaptation,” substantiated by the validation results for a host hospital. In the scenario, the host hospital was a small institution that may have not had an extensive data set of its own. The institution aimed to use an external data set, provided by a larger donor hospital, as a foundational training set. We assumed that the donor hospital provided its data set to the recipient hospital in an encrypted state. Moreover, we gradually increased the size of the host hospital’s data set when training the predictive model to ascertain the volume at which the utilization of its data—when adapting the donated data set as a foundational training set—became effective. The approach was applied to the comprehensive AMC data set, which acted as a donor hospital, on postoperative 30-day mortality; that is, we initiated our study with all the AMC data set and progressively incorporated an increasing proportion of the EUMC and SNUH data sets. EUMC is assumed to be the AMC data’s recipient. The AMC provides its own data in an encrypted state to augment the EUMC data set. Thereafter, the encrypted merged data set is used to train a logistic regression model, and inferences are made based on the EUMC’s plaintext data. Over the course of this study, the EUMC data set’s volume was incrementally increased by steps of 1000, 2000, 3000, and 4000, resulting in the sizes of 0, 1000, 3000, 6000, 10,000, 15,000, and 20,000. The adaptation process was applied with each increase in data size and integrated with the AMC data set, followed by training of the model and a thorough examination of the ensuing performance metrics—the receiver operating characteristic (AUROC) and area under the precision-recall curve (AUPRC). Moreover, we applied the same experimental process to the SNUH data set, using all the AMC data and gradually increasing the former’s proportion.

### Model Validation

Secure logistic regression with L2 regularization (called ridge regression) was developed using all the possible combinations of multicenter datasets with all input variables. The predictive performances were compared by assessing both the areas under the AUROC and AUPRC; the comparison was performed both numerically and statistically. Furthermore, AUROC and AUPRC were compared using the DeLong test [16].

### Statistical Analysis and Modeling Tools

Continuous variables were expressed as mean (SD), while categorical variables were expressed as count and percentage. Continuous and categorical variables were compared among the 3 institutions using one-way ANOVA and chi-squared test, respectively. Variables with 2-tailed *P* values <.05 were considered to hold statistical significance.

We comparatively analyzed feature importance for each institution’s data—as represented by the Shapley additive explanations (SHAP) values within logistic regression models—to investigate potential heterogeneity in data distributions across the 3 hospitals under consideration. Feature importance was evaluated using a logistic regression model and performed on plaintext data at each hospital without using HE. Statistical analyses were performed using Python 3.8.16 [17]. The DeLong test was performed using R 4.2.2 [18]. Secure logistic regression was conducted using scikit-learn 1.2.0 [19] and HEaaN.stat 0.2.0 [20].

## Results

### Study Population Characteristics

The average age of surgical patients at EUMC was the lowest, compared to the other 2 institutions, at 48.5 years (Table 1). Emergency surgeries occurred most frequently at the SNUH, with a rate of 7.4% (Table 1). Postoperative mortality within 30 days was relatively rare across all hospitals, with rates ranging from 0.2% to 0.4% (Table 1). Specifically, the rates were 0.3% at SNUH, 0.2% at AMC, and 0.2% at EUMC. The data characteristics of each hospital are shown in Table 1. When examining the SHA*P* values across all hospitals, we observed significant variation in data distributions, suggesting inherent biases within each hospital’s data set, as presented in Figure S4 in Multimedia Appendix 1. Figure S5 in Multimedia Appendix 1 presents the relative odds ratios of the predictor variables affecting the outcome variable in the logistic regression models trained based on each hospital’s data set. These odds ratios offer insights into each predictor variable’s effect on the likelihood of the outcome and help interpret associations’ magnitude and direction. Evidently, the distribution of the odds ratios of the predictor variables affecting the outcome variable differs among institutions. In the EUMC data set, only the age and general surgery department variables are statistically significant. In contrast, the significance of these and other variables varies across institutions, as demonstrated by the diverse distribution of odds ratios affecting the outcome variable depicted in Figure S2 in Multimedia Appendix 1.

**Table 1:** Data characteristics of the 3 medical institutions.

		SNUH^a^ (n=46,956)	AMC^b^ (n=162,184)	EUMC^c^ (n=131,867)	*P* value
**Demographic data**					
	Age (years), mean (SD)	55.9 (16.1)	54.2 (15.9)	48.5 (17.1)	<.001
	Sex (female), n (%)	26,236 (55.9)	94,413 (58.2)	79,232 (60.1)	<.001
	BMI (kg/m^2^), mean (SD)	24.6 (3.9)	24.2 (3.7)	23.8 (3.8)	<.001
**Preoperative laboratory results, mean (SD)**					
	White blood cell (10^3^/μL)	6.6 (3.0)	6.7 (2.4)	7.5 (3.9)	<.001
	Hemoglobin (g/dL)	13.1 (1.8)	12.8 (1.9)	13.1 (1.9)	<.001
	Platelet (10^3^/μL)	239.8 (73.5)	247.1 (72.7)	245.6 (72.0)	<.001
	Sodium (mmol/L)	140.2 (2.7)	139.8 (2.4)	140.7 (3.0)	<.001
	Potassium (mmol/L)	4.2 (0.4)	4.3 (0.3)	4.2 (0.4)	<.001
	BUN^d^ (mg/dL)	15.5 (8.1)	14.8 (6.8)	13.7 (6.9)	<.001
	Creatinine (mg/dL)	1.0 (1.1)	0.9 (0.7)	0.9 (0.7)	<.001
	Albumin 9g/dL)	4.2 (0.5)	3.8 (0.5)	4.1 (0.6)	<.001
	GOT^e^ (IU/L)	24.4 (36.7)	25.0 (33.7)	26.5 (95.0)	<.001
	GPT^f^ (IU/L)	23.4 (32.5)	22.7 (32.3)	25.1 (50.9)	<.001
	Glucose (mg/dL)	110.8 (30.5)	113.3 (36.9)	198.3 (243.9)	<.001
	PT^g^ (INR^h^)	1.0 (0.1)	1.0 (0.1)	1.0 (0.4)	<.001
	aPTT^i^ (s)	31.6 (4.6)	27.0 (3.3)	26.9 (5.4)	<.001
**Type of surgery, n (%)**					
	General surgery	13,487 (28.7)	60,6130 (36.4)	40,611 (30.8)	<.001
	Otolaryngologic surgery	4537 (9.7)	15,270 (10.8)	14,279 (10.8)	<.001
	Urologic surgery	4738 (10.1)	20,551 (12.7)	9117 (6.9)	<.001
	Orthopedic surgery	6736 (14.3)	24,480 (14.7)	23,486 (17.8)	<.001
	Gynecological surgery	4956 (14.5)	31,691 (19.5)	26,509 (20.1)	<.001
	Plastic surgery	1862 (4.0)	6213 (1.4)	9788 (7.4)	<.001
**Type of** **anesthesia, n (%)**					
	General anesthesia	36,060 (76.8)	149,542 (92.2)	100,223 (76.0)	<.001
	Neuroaxial anesthesia	5052 (16.5)	11,281 (7.0)	10,716 (8.1)	<.001
	MAC^j^	5792 (12.3)	0 (0.0)	4985 (3.8)	<.001
	Regional anesthesia	52 (0.1)	1361 (0.8)	509 (0.4)	<.001
	Emergency surgery	3456 (7.4)	8876 (5.5)	4208 (3.2)	<.001
	30-day mortality	156 (0.3)	306 (0.2)	316 (0.2)	<.001

^a^SNUH: Seoul National University Hospital.

^b^AMC: Asan Medical Center.

^c^EUMC: Ewha Womans University Medical Center.

^d^BUN: blood urea nitrogen.

^e^GOT: glutamate oxaloacetate transaminase.

^f^GPT: glutamate pyruvate transaminase.

^g^PT: prothrombin time.

^h^INR: international normalized ratio.

^i^aPTT: activated partial thromboplastin time.

^j^MAC: monitored anesthesia care.

### Data Preprocessing: Missing Value Characteristics and Standardization

Herein, the average rates of missing values were 0.00% to 7.63% for various features (Table S4 in Multimedia Appendix 1). This discrepancy—particularly in EUMC data for BMI, type of anesthesia, and preoperative glucose—may reflect distinct characteristics inherent to the databases of each hospital (refer to Figure S2 in Multimedia Appendix 1). There was a substantial correlation (absolute correlation value of 0.7 or greater) between variables that were part of collective testing procedures, such as laboratory tests. Conversely, the correlation between other variables was relatively weak, with absolute correlation values below 0.7 (Figure S1 in Multimedia Appendix 1). Variables with a higher incidence of missing values, such as BMI and type of anesthesia at EUMC, did not exhibit significant correlations with the missing values in other variables (absolute correlation value <0.7). The analysis did not reveal any consistent pattern in the occurrence of missing values across the hospitals, implying a random nature of missing data for individual patients at each facility (Figure S2 in Multimedia Appendix 1). Considering this randomness and the low intervariable correlation of missing values, we opted to impute the missing data using the respective variables’ median values.

### Model Performance

Table 2 presents the validation results of the bootstrapping performance of the secure logistic regression model using various single- and multicenter combinations. Typically, the AMC and EUMC models that already had abundant data delivered superior performance when externally validated using data from other institutions. However, regarding the AMC data set’s internal validation, the merged model using the entire data set demonstrated the highest performance, as indicated by AUROC of 0.941. Similarly, the AUPRC signified peak performance in the AMC data set’s internal validation when the model merged with the AMC and SNU data sets, reaching 0.132. Figure S3 in Multimedia Appendix 1 provides a comparative visualization of AUROC and AUPRC. Table S3 in Multimedia Appendix 1 presents *P* values, indicative of statistical significance via the DeLong test, when comparing the predictive performance of the plaintext single-institution model and encrypted multi-institution model based on AUROC and AUPRC. Small *P* values signify a statistically significant difference in the 2 models’ predictive performance.

**Table 2:** Validation results of single and merged secure logistic regression models for postoperative 30-day mortality on AMC, EUMC, and SNUH test data sets.

Train			Test				
			AMC^a^ (n=32,437)		SNUH^b^ (n=9392)		EUMC^c^ (n=26,373)
**Single (plaintext)**							
	AMC, mean AUROC^d^ (95% CI)	0.939 (0.927-0.950)		0.915 (0.902-0.928)		0.890 (0.867-0.912)	
	SNUH, mean AUROC (95% CI)	0.925 (0.913-0.936)		*0.942 (0.9300.953)* ^e^		0.937 (0.926-0.947)	
	EUMC, mean AUROC (95% CI)	0.880 (0.853-0.906)		0.906 (0.890-0.921)		0.952 (0.943-0.961)	
**Merged (ciphertext)**							
	AMC + EUMC, mean AUROC (95% CI)	0.931 (0.914-0.947)		0.919 (0.907-0.931)		0.952 (0.942-0.962)	
	AMC + SNUH, mean AUROC (95% CI)	0.940 (0.925-0.955)		0.927 (0.902-0.952)		0.934 (0.920-0.947)	
	SNUH + EUMC, mean AUROC (95% CI)	0.931 (0.916-0.945)		0.925 (0.903-0.946)		0.956 (0.950-0.962)	
	AMC + SNUH + EUMC, mean AUROC (95% CI)	*0.941 (0.927-0.955)*		0.929 (0.905-0.953)		*0.957 (0.951-0.963)*	
**Single (plaintext)**							
	AMC, mean AUPRC^f^ (95% CI)	0.125 (0.088-0.161)		0.089 (0.071-0.107)		0.072 (0.051-0.093)	
	SNUH, mean AUPRC (95% CI)	0.070 (0.044-0.095)		0.123 (0.099-0.146)		0.060 (0.075-0.072)	
	EUMC, mean AUPRC (95% CI)	0.087 (0.055-0.118)		0.085 (0.066-0.104)		*0.120 (0.090-0.150)*	
**Merged (ciphertext)**							
	AMC + EUMC, mean AUPRC (95% CI)	0.107 (0.078-0.136)		0.089 (0.074-0.104)		0.107 (0.081-0.133)	
	AMC + SNUH, mean AUPRC (95% CI)	*0.132 (0.094-0.169)*		*0.171 (0.112-0.230)*		0.081 (0.060-0.102)	
	SNUH + EUMC, mean AUPRC (95% CI)	0.098 (0.069-0.126)		0.098 (0.069-0.126)		0.116 (0.089-0.143)	
	AMC + SNUH + EUMC, mean AUPRC (95% CI)	0.113 (0.082-0.144)		0.113 (0.082-0.144)		0.114 (0.089-0.139)	

^a^AMC: Asan Medical Center.

^b^SNUH: Seoul National University Hospital.

^c^EUMC: Ewha Womans University Medical Center.

^d^AUROC: area under the receiver operating characteristic curve.

^e^Italics indicate significant results.

^f^AUPRC: area under the precision-recall curve.

For unencrypted data, we used a plaintext version of the logistic regression model, developed from scratch using NumPy and featuring an architecture identical to that of the HE-based logistic regression model. Further, we evaluated the discrepancies between the results computed in ciphertext and subsequently decrypted, compared to those calculated directly in plaintext. The mean absolute difference was 2.02×10^–5^, indicating a marginal difference. The minimum absolute difference was remarkably low at 6.56×10^–10^, while the maximum absolute difference reached 7.71×10^–4^. This observation suggests that the outcomes—irrespective of whether they are computed in plaintext or ciphertext—demonstrate an almost indistinguishable difference.

### Model Adaptation Results

**Figure 2:**
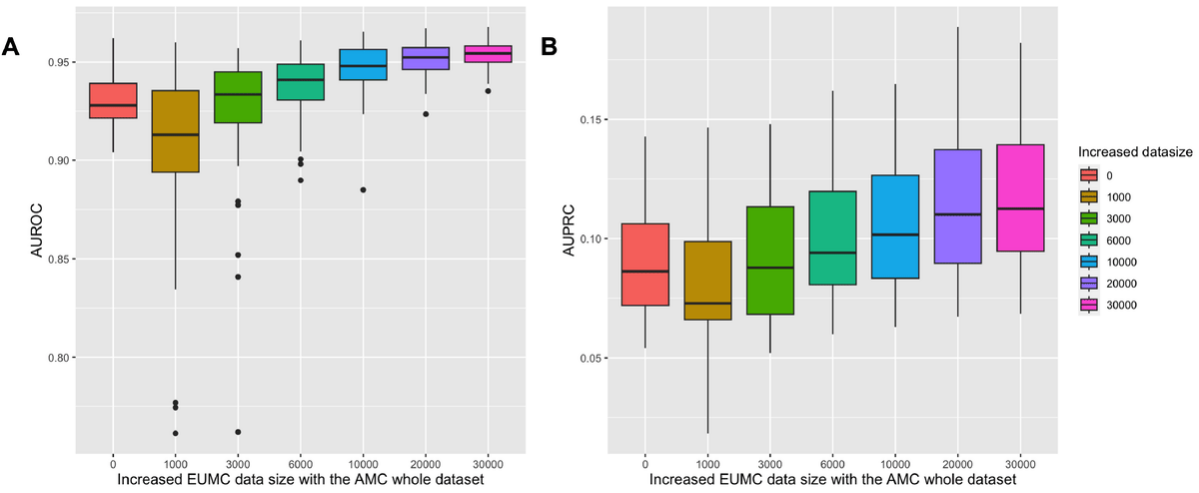
Validation results of bootstrap samples using increased EUMC data size with the AMC whole data set for postoperative 30-day mortality. (a) Boxplot analysis of AUROC using bootstrap samples; (b) boxplot analysis of AUPRC using bootstrap samples. AMC: Asan Medical Center; AUPRC: area under the precision-recall curve; AUROC: area under the receiver operating characteristic curve; EUMC: Ewha Womans University Medical Center; SNUH: Seoul National University Hospital.

**Figure 3:**
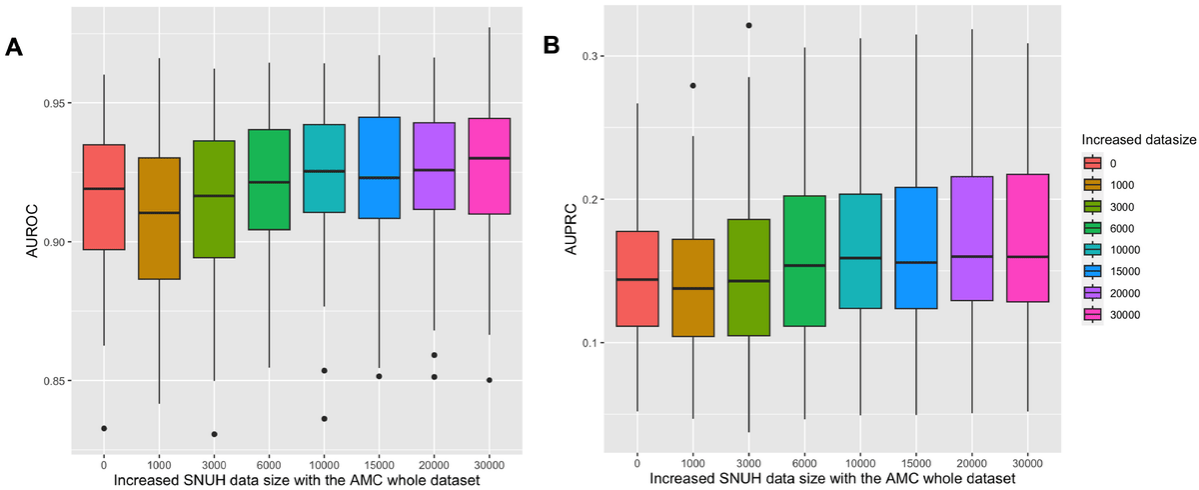
Validation results of bootstrap samples using increased SNUH data size with the AMC whole data set for postoperative 30-day mortality. (a) Boxplot analysis of AUROC; (b) boxplot analysis of AUPRC. AMC: Asan Medical Center; AUPRC: area under the precision-recall curve; AUROC: area under the receiver operating characteristic curve; EUMC: Ewha Womans University Medical Center; SNUH: Seoul National University Hospital.

We investigated the scenario of model adaptation wherein we gradually incorporated the data set from another institute. Using the EUMC data set, starting with an AUROC of 0.930, there was an initial temporary decline to 0.906 when the first 1000 records from the EUMC data set were incorporated into the complete AMC data set (Figure 2, Table S2A in Multimedia Appendix 1). As more EUMC records were progressively added, a significant improvement in AUROC was observed, eventually reaching 0.954 (Figure 2, Table S2A in Multimedia Appendix 1). Similarly, the AUPRC initially decreased from 0.09 to 0.075 with the addition of the initial 1000 EUMC data to the total AMC data (Figure 2, Table S2A in Multimedia Appendix 1). However, as we continued introducing more EUMC data, the AUPRC began increasing (Figure 2, Table S2A in Multimedia Appendix 1). Upon the inclusion of 30,000 EUMC records, the AUPRC ascended to 0.11 (Figure 2, Table S2A in Multimedia Appendix 1). Using the SNUH data set, we began with an AUROC of 0.916. The increase was less pronounced than that observed with the EUMC data set (Figure 3, Table S2B in Multimedia Appendix 1). However, as we progressively included segments of the SNUH data set, the AUROC exhibited a moderate trend of progressive improvement, eventually reaching 0.926 (Figure 3, Table S2B in Multimedia Appendix 1). A decrease in the AUPRC from 0.151 to 0.131 was observed when the first 1000 SNUH data were added to the AMC data set (Figure 3, Table S2B in Multimedia Appendix 1). As more SNUH data were added, the AUPRC gradually increased, improving to approximately 0.149, compared to the AMC single-institution model’s performance, once 30,000 data points were included (Figure 3, Table S2B in Multimedia Appendix 1).

## Discussion

### Overview of Multi-Institutional Model Performance and Implications

This study demonstrated the potential of overcoming limitations associated with single-institution models, such as reduced external predictive power and data overfitting, through secure multi-institutional data integration using HE technology. Our approach effectively adapts predictive models to specific hospital environments, indicating a substantial improvement in model performance across different data sets. The results suggest that small- and medium-sized hospitals with limited data can enhance the predictive performance of their AI models by adopting data from larger hospitals and conducting additional combined learning using HE technology. The significance of this study lies in its practical application and validation of HE technology using real-world, multi-institutional clinical data, laying the groundwork for its potential applicability to various forms of multi-institutional clinical data in future research.

### Advantages and Challenges of Multicenter Studies

The transition from single-center to multicenter studies generates large data sets (“big data”), enhancing the robustness and generalizability of research findings. These larger and more diverse data sets increase the accuracy and applicability of the results. However, multicenter studies introduce challenges such as secure and legal data sharing, inherent incompatibility between data security and research efficacy, and potential biases from selective participant inclusion [21]. To address these issues, researchers are exploring innovative PETs like HE, federated learning, and multiparty computation, which enable secure data analyses while preserving patient confidentiality.

### Federated Learning and Comparison to HE

Federated learning has been proposed as a security solution in multi-institutional data environments, as it only shares each local model’s weights or parameters. The strength of federated learning—a more decentralized approach than ours—is that no patient-level data are transferred to third parties with or without encryption. However, even with aggregate-level data, such as weights of a model, patient-level information can potentially be inferred [22-25].

In this multicenter study, we used cutting-edge HE to protect personal information leakage and data security. Additionally, HE enables operations and predictive modeling on encrypted data, providing an ultimate solution that can completely resolve issues related to personal information leakage and data security. Furthermore, HE provides the maximum (strongest) security when used appropriately, such as in outsourced computation, wherein HE secures data breaches in computation. In medical fields, HE has been applied to numerous cases for fulfilling privacy requirements [26]. Previous computational inefficiency of HE may have limited its application in computation-intensive steps, such as in developing a prediction model; however, recent advancements have led the technology to be used in practice. The present multicenter study demonstrated that a prediction model can be developed completely without a data breach risk in training using HE.

### Limitations and Future Directions

While HE technology allowed secure data integration, it introduced several challenges. Notably, encrypting data led to a marked increase in data size compared to plaintext, intensifying data storage requirements. Additionally, the encrypted model necessitated longer training time. Furthermore, in multi-institutional contexts, such as health care data sharing, key management in multiparty HE becomes a complex, practical challenge. These factors—expanded data size, prolonged training periods, and intricacies of key management—are essential considerations in the effective deployment and ongoing development of secure logistic regression models within encrypted data frameworks.

The study also highlighted limitations in predictive performance when models trained on diverse data sets were applied to individual hospitals. In some data sets, the merged data model's predictive performance fell short of the single-institution data model. This discrepancy indicates a complex interplay between data heterogeneity and model performance, suggesting that predictive performance may not always be enhanced through data augmentation alone, as evidenced in this study. A prediction model may lose prediction power in some institutions when trained using data from institutions with disparate data distributions. Consequently, when implementing a trained model on individual hospital data, we occasionally observed a deviation from our initial expectation that a model trained on the merged set would invariably outperform others.

Another limitation of the study was the reliance on retrospectively collected data, featuring varying extraction periods across institutions. The effects of temporal changes, including advancements in medical technology, were not fully adjusted for, potentially reducing the results' discernibility.

To address these limitations, future research should explore methods for data integration that adjust for heterogeneity. This can be achieved by prospectively collecting data from multiple institutions and conducting comparative studies on predictive performance using HE technology. Such methodologies would help to mitigate the impact of varying data extraction periods and temporal changes in medical technology. Additionally, the use of advanced statistical methods to better account for data heterogeneity might be explored as a promising avenue for further research. These studies would undoubtedly offer invaluable insights into potential strategies for enhancing predictive performance in multi-institutional settings while preserving data privacy and security.

### Conclusions

In conclusion, this study demonstrated the practicality of using HE technology to combine data from real-world multi-institutional sources and develop predictive models for in-hospital mortality within 30 days postoperatively. Additionally, we showcased the implementation of privacy-preserving artificial intelligence prediction models. The findings highlight the potential for both practical applications and protection of personal information in the realm of predictive modeling. HE technology should be applied to diverse forms of multi-institutional clinical data in future endeavors to replicate, validate, and extend this study’s findings.

## Appendix

Multimedia Appendix 1 Supplementary material.

## Abbreviations

AMC: Asan Medical Center
AUPRC: area under the precision-recall curve
AUROC: area under the receiver operating characteristic curve
DB: database
EUMC: Ewha Womans University Medical Center
HE: homomorphic encryption
PETs: privacy-enhancing technologies
SHAP: shapley additive explanations
SNUH: Seoul National University Hospital

## Data Availability

The data set used in the study is not publicly available. However, it can be provided upon reasonable request to the corresponding authors. For the SNUH data set, we used the deidentified open data set, INSPIRE, which was compiled and made publicly available by SNUH. The construction and release of the INSPIRE data set were separately approved by the institutional review board of SNUH (H-2210-078-1368). The codes that support the findings of the study are available for download on GitHub [27].
