# Supplementary material for: Privacy-Preserving Prediction of Postoperative Mortality in Multi-Institutional Data: Development and Usability Study
Source: JMIR Med Inform. 2024 Jul 5;12:e56893. doi: 10.2196/56893 (PMC11259763; doi:10.2196/56893)
Supplement: Multimedia Appendix 1 [file medinform_v12i1e56893_app1.docx]

**Supplementary material**

**Privacy-Preserving Prediction of Postoperative Mortality in Multi-Institutional Data**

Jungyo Suh, MD^1^**^†^**; Garam Lee, MS^2^**^†^**; Jung Woo Kim, PhD^2^; Junbum Shin, PhD^2^; Yi-Jun Kim, MD, PhD^3^; Sang-Wook Lee, MD, PhD;^4^*; Sulgi Kim, PhD^2^*

**Contents**

**Supplementary methods.**

**Table S1. Missing value characteristics**

**Table S2. (A) Validation results of trained models using increased EUMC data size with the AMC whole dataset on the AMC, EUMC, and SNUH test datasets**

**Table S2. (B) Validation results of trained models using increased SNUH data size with the AMC whole dataset on the AMC, EUMC, and SNUH test datasets**

**Table S3. (A) Comparison of AUROC between single and merge-trained models for postoperative 30-day mortality in AMC, EUMC, and SNUH test datasets**

**Table S3. (B) Comparison of AUPRC between single and merge-trained models for postoperative 30-day mortality in the AMC, EUMC, and SNUH test datasets**

**Figure S1. SHAP value summary plots generated from the logistic regression models of each hospital's dataset after preprocessing**

**(A) SHAP value summary plot for SNUH**

**(B) SHAP value summary plot for AMC**

**(C) SHAP value summary plot for EUMC**

**Figure S2.** **Odds ratio plots for logistic regression models**

**(A) Odds ratio plot for SNUH**

**(B) Odds ratio plot for AMC**

**(C) Odds ratio plot for EUMC**

**Figure S3. (A) AUROC and AUPRC of single (unencrypted) and all possible merged (encrypted) models for postoperative 30-day mortality in the AMC dataset**

**Figure S3. (B) AUROC and AUPRC of single (unencrypted) and all possible merged (encrypted) models for postoperative 30-day mortality in the EUMC dataset**

**Figure S3. (C) AUROC and AUPRC of single (unencrypted) and all possible merged (encrypted) models for postoperative 30-day mortality in the SNUH dataset**

**Figure S4. Distribution of missing values and data completeness of dataset in each hospital**

**Figure S5. Nullity correlation heatmap in each hospital**

**Supplementary methods.**

**Technical Overview of Homomorphic Encryption**

This study adopts the CKKS homomorphic encryption (HE) scheme that supports approximate arithmetic operations over encrypted real-valued vectors. While other HE schemes can be applied for computations over integers, the CKKS scheme allows for encrypted computations over real and complex numbers, greatly increasing its real-world applications, including machine learning and data analysis. CKKS is a leveled HE scheme with a unique operation called bootstrapping that addresses the issue of level reduction, allowing for a theoretically unlimited number of computations by continually refreshing the ciphertext's level.

The CKKS scheme encrypts multiple complex numbers into a single ciphertext and supports Single Instruction Multiple Data (SIMD) operations. A notable feature of the CKKS scheme is its operation on both ciphertexts and plaintexts, providing flexibility for various user scenarios while also reducing computational noise. This study employs HE parameters that ensure a 128-bit security level, providing secure protection for the homomorphically encrypted ciphertext.

A secure logistic regression model, widely used in fields like machine learning and biomedicine, was applied using HE to maintain data privacy. In a scenario where a small institution incorporates data from larger institutions, the donor institutions can provide their data encrypted using HE, ensuring the privacy of their data while also facilitating model adaptation at the recipient institution.

In single-institutional training, the use of cryptographic methods may be unnecessary. However, in multi-institutional settings, HE becomes pivotal in maintaining privacy while sharing data. We compared the performance of an encrypted model (ciphertext) and an unencrypted model (plaintext) by implementing the same logistic regression model. Our HE-based logistic regression employs Nesterov's accelerated gradient (NAG) optimizer, which is compatible with HE operations and guarantees faster convergence.

Owing to the types of available operations on HE, the sigmoid function of logistic regression is approximated by a polynomial function. Despite this, our HE-friendly secure logistic regression, where training, inference, and evaluation are all performed in an encrypted state, maintains comparable performance to standard logistic regression models. This demonstrates the potential of HE in facilitating secure multi-institutional collaboration while preserving data privacy.

**HE Library: HEaaN**

The HEaaN library—a C++ based resource instrumental in facilitating HE, decryption, and key generation processes—supports fundamental homomorphic evaluations, including addition, multiplication, and rotation, adhering to the CKKS scheme. We executed data encryption using version 0.2.4 of the HEaaN library, with the FGb parameter set to a ciphertext dimension of 2^16. Further, the HEaaN-SDK, a Python library, is tailored for data science, data analysis, and the implementation of machine learning models under HE, utilizing the capabilities of the HEaaN library. To conduct logistic regression on encrypted data, an HE-based version of the method is required. Thus, we employed HEaaN-SDK version 0.2.1.

**Table S1. Missing value characteristics**

| Characteristics | SNUH (n = 46,956) | | |  | AMC (n = 162,184) | | |  | EUMC (n = 131,867) | | |
| --- | --- | --- | --- | --- | --- | --- | --- | --- | --- | --- | --- |
|  | Available data | Missing data | Missing data (%) |  | Available data | Missing data | Missing data (%) |  | Available data | Missing data | Missing data (%) |
| Demographics |  |  |  |  |  |  |  |  |  |  |  |
| Age | 46,956 | 0 | 0.00 |  | 162,184 | 0 | 0.00 |  | 131,867 | 0 | 0.00 |
| Sex | 46,956 | 0 | 0.00 |  | 162,184 | 0 | 0.00 |  | 131,867 | 0 | 0.00 |
| Body mass index | 46,365 | 591 | 1.26 |  | 149,804 | 12,380 | 7.63 |  | 53,819 | 78,048 | 59.19 |
| Preoperative laboratory tests |  |  |  |  |  |  |  |  |  |  |  |
| White blood cells | 42,561 | 4,395 | 9.36 |  | 158,073 | 4,111 | 2.53 |  | 131,761 | 106 | 0.08 |
| Hemoglobin | 42,734 | 4,222 | 8.99 |  | 158,073 | 4,111 | 2.53 |  | 131,867 | 0 | 0.00 |
| Platelet | 42,490 | 4,466 | 9.51 |  | 158,073 | 4,111 | 2.53 |  | 131,757 | 110 | 0.08 |
| Prothrombin time | 42,233 | 4,723 | 10.06 |  | 151,590 | 10,594 | 6.53 |  | 116,473 | 15,394 | 11.67 |
| Activated partial thromboplastin time | 42,090 | 4,866 | 10.36 |  | 150,901 | 11,283 | 6.96 |  | 116,945 | 14,922 | 11.32 |
| Sodium | 42,596 | 4,360 | 9.29 |  | 158,559 | 3,625 | 2.24 |  | 123,399 | 8,468 | 6.42 |
| Potassium | 42,591 | 4,365 | 9.30 |  | 158,562 | 3,622 | 2.23 |  | 123,400 | 8,467 | 6.42 |
| Blood urea nitrogen | 42,226 | 4,730 | 10.07 |  | 153,369 | 8,815 | 5.44 |  | 127,193 | 4,674 | 3.54 |
| Creatinine | 46,477 | 479 | 1.02 |  | 157,792 | 4,392 | 2.71 |  | 127,394 | 4,473 | 3.39 |
| Aspartate transaminase | 42,146 | 4,810 | 10.24 |  | 158,198 | 3,986 | 2.46 |  | 127,541 | 4,326 | 3.28 |
| Alanine transaminase | 42,141 | 4,815 | 10.25 |  | 158,199 | 3,985 | 2.46 |  | 127,539 | 4,328 | 3.28 |
| Albumin | 42,358 | 4,598 | 2.53 |  | 158,081 | 4,103 | 2.53 |  | 108,198 | 23,669 | 17.95 |
| Surgical information |  |  |  |  |  |  |  |  |  |  |  |
| Emergency status of surgery | 46,956 | 0 | 0.00 |  | 160,330 | 1,854 | 1.14 |  | 131,867 | 0 | 0.00 |
| Department of Surgery | 46,956 | 0 | 0.00 |  | 162,184 | 0 | 0.00 |  | 116,434 | 15,434 | 11.70 |
| Type of anesthesia | 46,956 | 0 | 0.00 |  | 162,184 | 0 | 0.00 |  | 131,867 | 0 | 0.00 |
| Mean of missing data (%) |  |  | 3.76 |  |  |  | 5.30 |  |  |  | 16.33 |

SNUH, Seoul National University Hospital; AMC, Asan Medical Center; EUMC, Ewha Womans University Medical Center

**Table S2. (A) Validation results of trained models using increased EUMC data size with the AMC whole dataset on the AMC, EUMC, and SNUH test datasets**

| Center |  | Increased EUMC data size with the AMC whole dataset | | | | | |  |
| --- | --- | --- | --- | --- | --- | --- | --- | --- |
|  | 0 (AMC only) | 1,000 | 3,000 | 6,000 | 10,000 | 20,000 | 30,000 | |
| **AUROC** | | | | | | | |  |
| AMC | 0.939  (0.925–0.953) | 0.939  (0.925–0.954) | 0.940  (0.926–0.954) | 0.940  (0.926–0.954) | 0.940  (0.926–0.954) | 0.940  (0.927–0.955) | 0.941  (0.927–0.955) | |
| **EUMC** | 0.930  (0.918–0.942) | 0.906  (0.862–0.949) | 0.925  (0.891–0.959) | 0.937  (0.919–0.955) | 0.946  (0.933–0.960) | 0.951  (0.943–0.960) | 0.954  (0.947–0.961) | |
| SNUH | 0.916  (0.889–0.944) | 0.891  (0.858–0.923) | 0.894  (0.857–0.930) | 0.897  (0.863–0.930) | 0.902  (0.868–0.936) | 0.903  (0.870–0.935) | 0.907  (0.877–0.937) | |
| **AUPRC** | | | | | | | |  |
| AMC | 0.132  (0.094–0.169) | 0.131  (0.940–0.169) | 0.132  (0.067–0.170) | 0.131  (0.093–0.169) | 0.132  (0.094–0.170) | 0.133  (0.096–0.170) | 0.133  (0.094–0.170) | |
| **EUMC** | 0.090  (0.067–0.114) | 0.075  (0.047–0.104) | 0.091  (0.071–0.118) | 0.094  (0.073–0.114) | 0.102  (0.078–0.124) | 0.109  (0.084–0.129) | 0.110  (0.087–0.131) | |
| SNUH | 0.151  (0.099–0.203) | 0.144  (0.090–0.196) | 0.150  (0.092–0.207) | 0.161  (0.101–0.221) | 0.169  (0.109–0.229) | 0.172  (0.111–0.233) | 0.174  (0.113–0.235) | |

SNUH, Seoul National University Hospital; AMC, Asan Medical Center; EUMC, Ewha Womans University Medical Center.

**Table S2. (B) Validation results of trained models using increased SNUH data size with the AMC whole dataset on the AMC, EUMC, and SNUH test datasets**

| Center |  | Increased SNUH data size with the AMC whole dataset | | | | | |  |
| --- | --- | --- | --- | --- | --- | --- | --- | --- |
|  | 0 (AMC only) | 1000 | 3,000 | 6,000 | 10,000 | 20,000 | 30,000 | |
| **AUROC** | | | | | | | |  |
| AMC | 0.939  (0.925–0.953) | 0.940  (0.925–0.954) | 0.939  (0.926–0.953) | 0.941  (0.926–0.955) | 0.940  (0.926–0.954) | 0.940  (0.926–0.954) | 0.940  (0.927–0.954) | |
| EUMC | 0.930  (0.918–0.942) | 0.910  (0.878–0.941) | 0.910  (0.883–0.937) | 0.910  (0.886–0.933) | 0.913  (0.888–0.938) | 0.918  (0.892–0.945) | 0.927  (0.902–0.952) | |
| **SNUH** | 0.916  (0.889–0.944) | 0.908  (0.879–0.938) | 0.914  (0.885–0.942) | 0.920  (0.892–0.947) | 0.920  (0.893–0.948) | 0.923  (0.897–0.950) | 0.926  (0.899–0.950) | |
| **AUPRC** | | | | | | | |  |
| AMC | 0.132  (0.094–0.170) | 0.132  (0.93–0.170) | 0.131  (0.94–0.168) | 0.132  (0.095–0.169) | 0.133  (0.095–0.170) | 0.131  (0.093–0.169) | 0.128  (0.092–0.170) | |
| EUMC | 0.089  (0.066–0.113) | 0.081  (0.054–0.108) | 0.093  (0.066–0.121) | 0.100  (0.074–0.126) | 0.106  (0.079–0.132) | 0.112  (0.084–0.141) | 0.115  (0.087–0.143) | |
| **SNUH** | 0.151  (0.099–0.199) | 0.131  (0.083–0.179) | 0.134  (0.085–0.182) | 0.138  (0.087–0.190) | 0.139  (0.088–0.190) | 0.146  (0.093–0.199) | 0.149  (0.093–0.204) | |

SNUH, Seoul National University Hospital; AMC, Asan Medical Center; EUMC, Ewha Womans University Medical Center.

**Table S3. (A) Comparison of AUROC between single (unencrypted) and merge-trained (encrypted) models for postoperative 30-day mortality in the AMC, EUMC, and SNUH test datasets by DeLong test**

| **Trained models** | **AUROC for AMC test dataset** | | | | |
| --- | --- | --- | --- | --- | --- |
|  | **AMC** | **SNUH** | **EUMC** | **AMC + EUMC** | **AMC + SNUH** |
| **AMC** |  |  |  |  |  |
| **SNUH** | 0.140 |  |  |  |  |
| **EUMC** | 0.034 | 0.392 |  |  |  |
| **AMC + EUMC** | 0.086 | 0.456 | 0.045 |  |  |
| **AMC + SNUH** | 0.514 | 0.108 | 0.032 | 0.099 |  |
| **AMC + SNUH + EUMC** | 0.102 | 0.436 | 0.044 | 0.862 | 0.090 |
|  |  |  |  |  |  |
| **Trained models** | **AUROC for EUMC test dataset** | | | | |
|  | **AMC** | **SNUH** | **EUMC** | **AMC + EUMC** | **AMC + SNUH** |
| **AMC** |  |  |  |  |  |
| **SNUH** | 0.042 |  |  |  |  |
| **EUMC** | 0.484 | 0.094 |  |  |  |
| **AMC + EUMC** | 0.130 | 0.021 | 0.238 |  |  |
| **AMC + SNUH** | 0.334 | 0.022 | 0.289 | 0.042 |  |
| **AMC + SNUH + EUMC** | 0.109 | 0.016 | 0.301 | 0.936 | 0.031 |
|  |  |  |  |  |  |
| **Trained models** | **AUROC for SNUH test dataset** | | | | |
|  | **AMC** | **SNUH** | **EUMC** | **AMC + EUMC** | **AMC + SNUH** |
| **AMC** |  |  |  |  |  |
| **SNUH** | 0.941 |  |  |  |  |
| **EUMC** | 0.308 | 0.435 |  |  |  |
| **AMC + EUMC** | 0.682 | 0.822 | 0.064 |  |  |
| **AMC + SNUH** | 0.010 | 0.197 | 0.034 | 0.059 |  |
| **AMC + SNUH + EUMC** | 0.593 | 0.660 | 0.014 | 0.005 | 0.206 |

The results presented in the table are p-values, which represent the level of statistical significance when comparing the AUROC of each model, using the DeLong test. SNUH, Seoul National University Hospital; AMC, Asan Medical Center; EUMC, Ewha Womans University Medical Center; AUROC, area under the received operating curve

**Table S3. (B) Comparison of AUPRC between single (unencrypted) and merge-trained (encrypted) models for postoperative 30-day mortality in the AMC, EUMC, and SNUH test datasets by DeLong test**

| **Trained models** | **AUPRC for AMC test dataset** | | | | |
| --- | --- | --- | --- | --- | --- |
|  | **AMC** | **SNUH** | **EUMC** | **AMC + EUMC** | **AMC + SNUH** |
| **AMC** |  |  |  |  |  |
| **SNUH** | 0.056 |  |  |  |  |
| **EUMC** | 0.070 | 0.259 |  |  |  |
| **AMC + EUMC** | 0.191 | 0.589 | 0.901 |  |  |
| **AMC + SNUH** | 0.386 | 0.934 | 0.905 | 0.818 |  |
| **AMC + SNUH + EUMC** | 0.199 | 0.577 | 0.904 | 0.474 | 0.202 |
|  |  |  |  |  |  |
| **Trained models** | **AUPRC for EUMC test dataset** | | | | |
|  | **AMC** | **SNUH** | **EUMC** | **AMC + EUMC** | **AMC + SNUH** |
| **AMC** |  |  |  |  |  |
| **SNUH** | 0.896 |  |  |  |  |
| **EUMC** | 0.001 | 0.998 |  |  |  |
| **AMC + EUMC** | 0.820 | 0.964 | 0.587 |  |  |
| **AMC + SNUH** | 0.001 | 0.975 | 0.001 | 0.002 |  |
| **AMC + SNUH + EUMC** | 0.709 | 0.924 | 0.142 | 0.022 | 0.999 |
|  |  |  |  |  |  |
| **Trained models** | **AUPRC for SNUH test dataset** | | | | |
|  | **AMC** | **SNUH** | **EUMC** | **AMC + EUMC** | **AMC + SNUH** |
| **AMC** |  |  |  |  |  |
| **SNUH** | 0.896 |  |  |  |  |
| **EUMC** | 0.910 | 0.022 |  |  |  |
| **AMC + EUMC** | 0.306 | 0.083 | 0.995 |  |  |
| **AMC + SNUH** | 0.818 | 0.147 | 0.978 | 0.874 |  |
| **AMC + SNUH + EUMC** | 0.312 | 0.077 | 0.997 | 0.698 | 0.109 |

The results presented in the table are p-values, which represent the level of statistical significance when comparing the AUPRC of each model, using the DeLong test. SNUH, Seoul National University Hospital; AMC, Asan Medical Center; EUMC, Ewha Womans University Medical Center; AUPRC, area under the precision–recall curve.

**Figure S1. SHAP value summary plots generated from the logistic regression models of each hospital's dataset after preprocessing. The model was trained on 80% of the data, with the remaining 20% used as a test set for generating these SHAP values.**

**(A) SHAP value summary plot for SNUH**


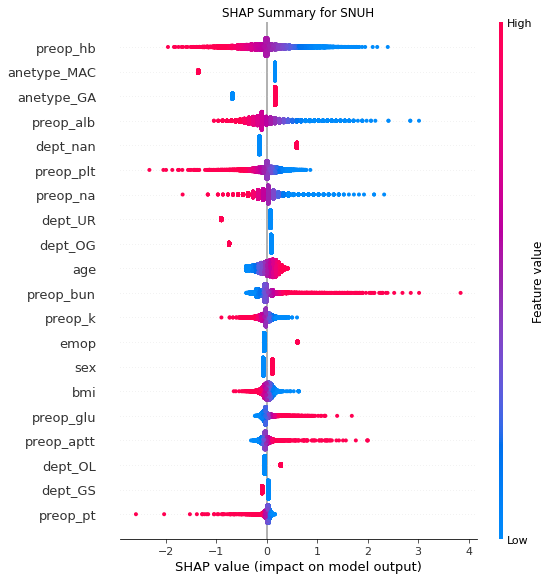


**
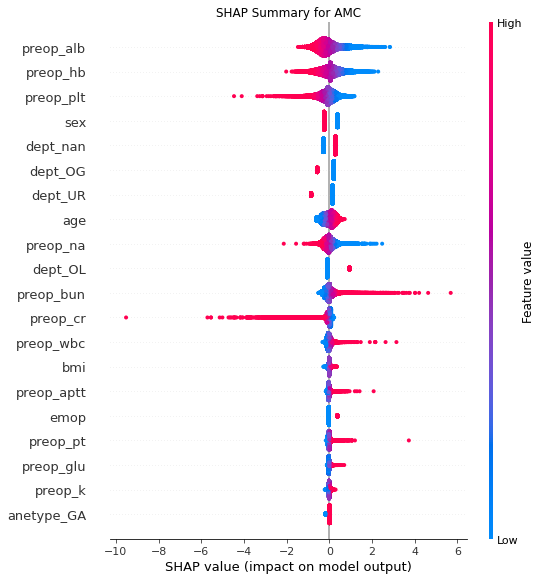
(B) SHAP value summary plot for AMC**

**(C) SHAP value summary plot for EUMC**

Shapley additive explanations, SHAP; SNUH, Seoul National University Hospital; AMC, Asan Medical Center; EUMC, Ewha Womans University Medical Center.
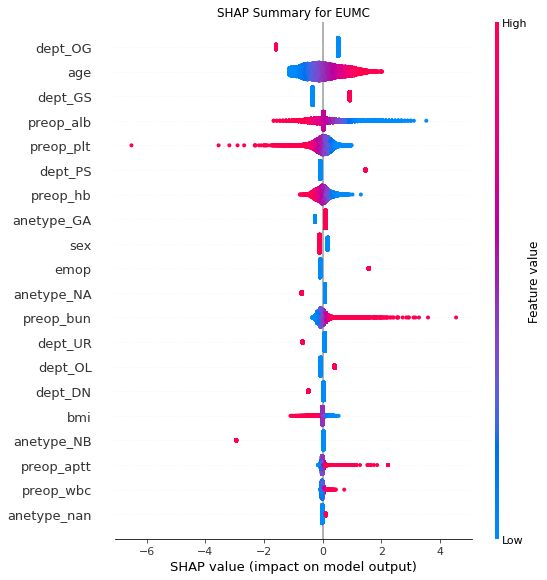


**Figure S2.** Odds ratio plots for logistic regression models. The odds ratio plots depict the relative odds of the outcome variable based on the logistic regression models trained on each hospital's dataset.

**(A)** Odds ratio plot for SNUH


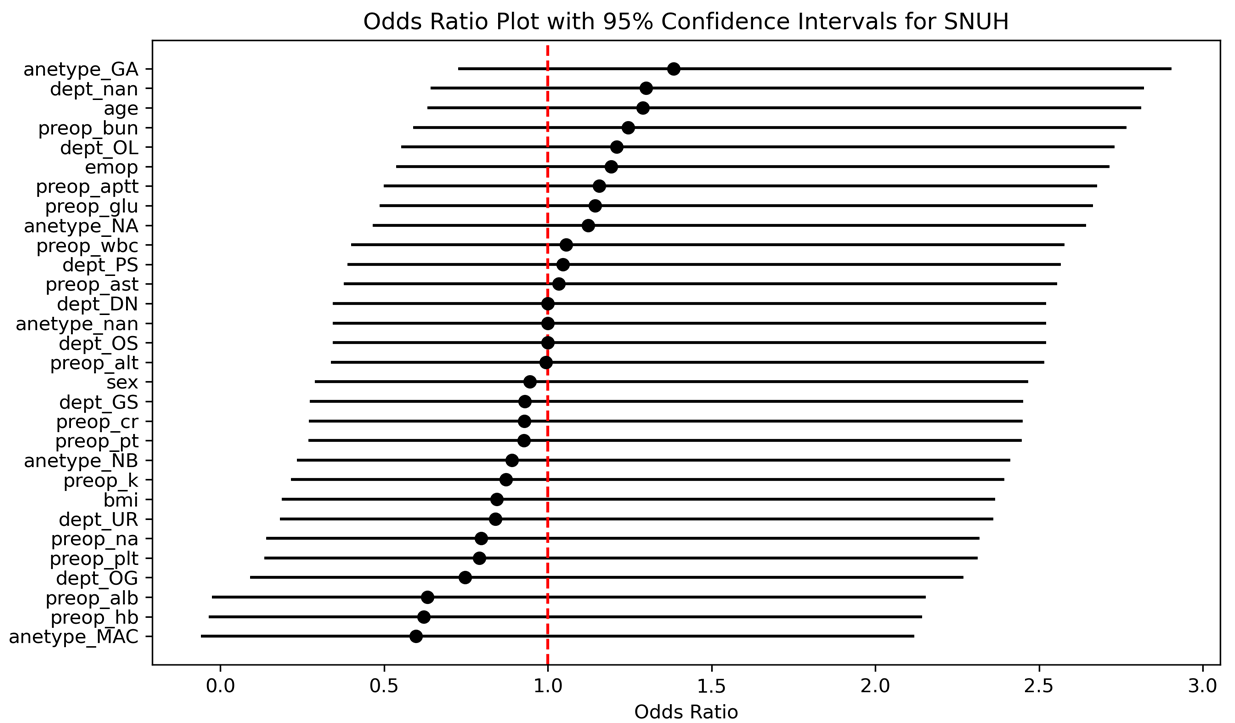


**(B)** Odds ratio plot for AMC


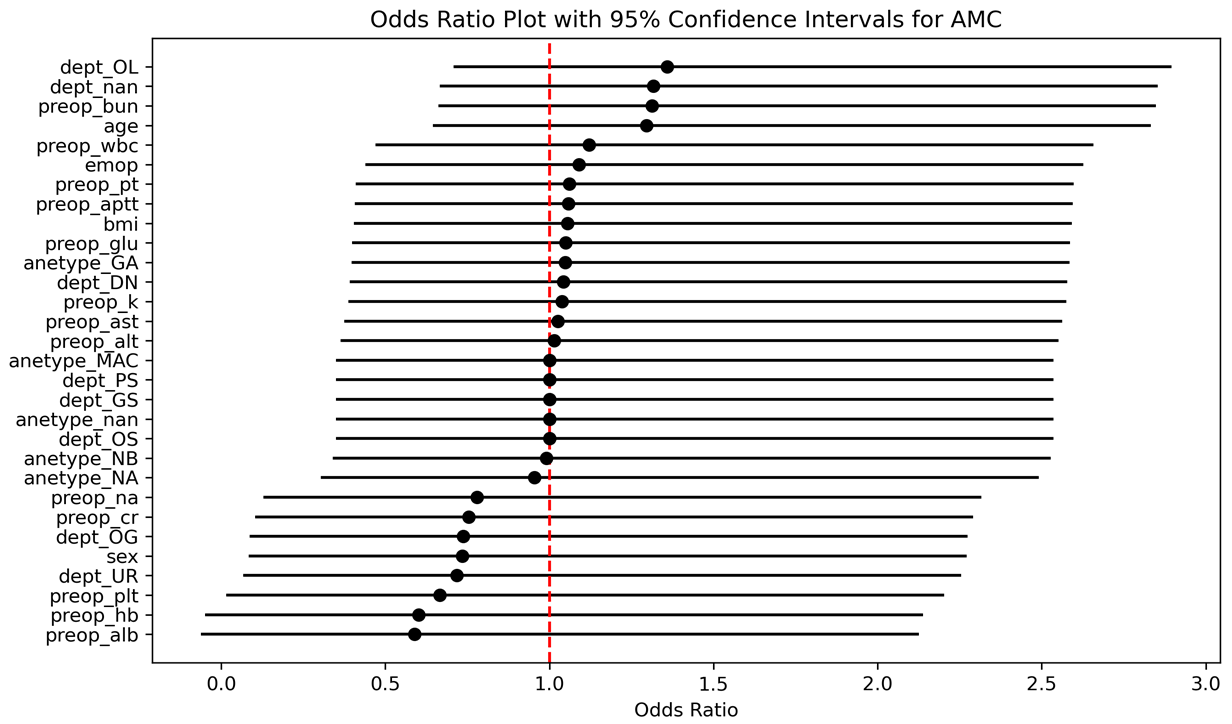


**(C)** Odds ratio plot for EUMC


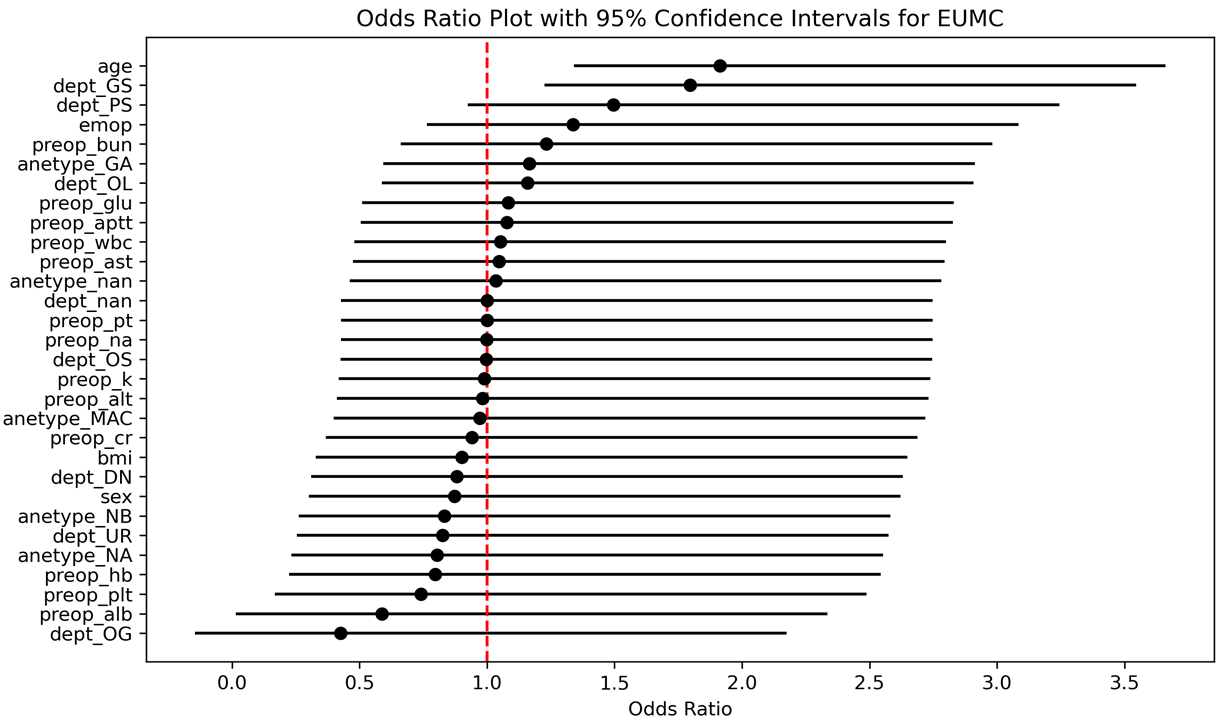


**Figure S3. (A) AUROC and AUPRC of single (unencrypted) and all possible merged (encrypted) models for postoperative 30-day mortality in the AMC dataset**


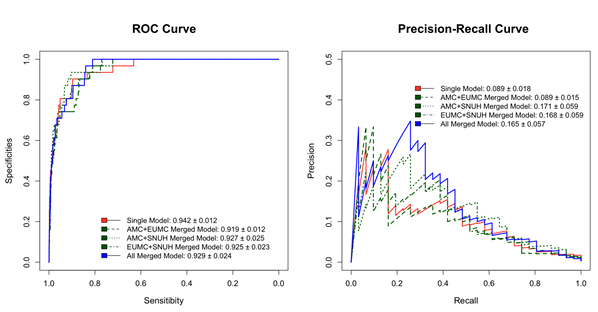


SNUH, Seoul National University Hospital; AMC, Asan Medical Center; EUMC, Ewha Womans University Medical Center; AUROC, area under the received operating curve; AUPRC, area under the precision–recall curve.

**(B) AUROC and AUPRC of single (unencrypted) and all possible merged (encrypted) models for postoperative 30-day mortality in the EUMC dataset**


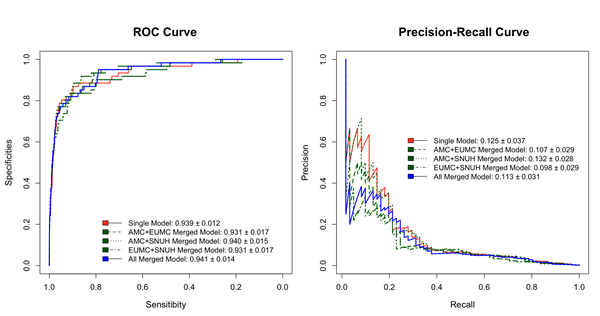


SNUH, Seoul National University Hospital; AMC, Asan Medical Center; EUMC, Ewha Womans University Medical Center; AUROC, area under the received operating curve; AUPRC, area under the precision–recall curve.

**(C) AUROC and AUPRC of single (unencrypted) and all possible merged (encrypted) models for postoperative 30-day mortality in the SNUH dataset**


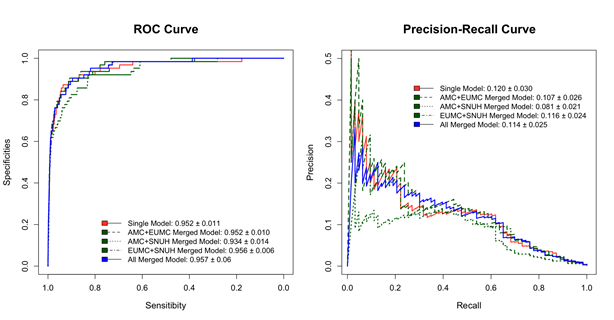


SNUH, Seoul National University Hospital; AMC, Asan Medical Center; EUMC, Ewha Womans University Medical Center; AUROC, area under the received operating curve; AUPRC, area under the precision–recall curve.

**Figure S4. Distribution of missing values and data completeness of datasets in each hospital**


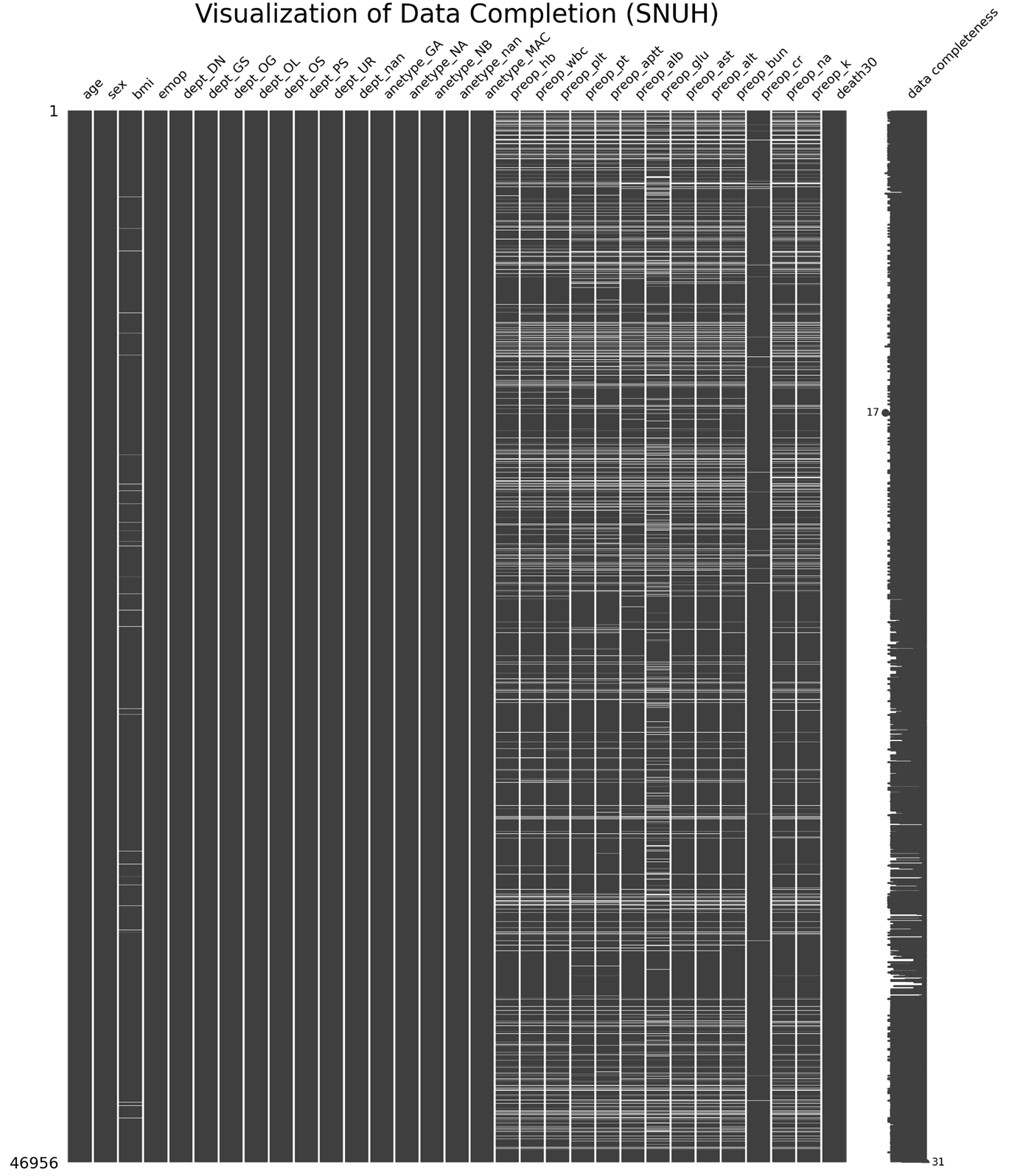


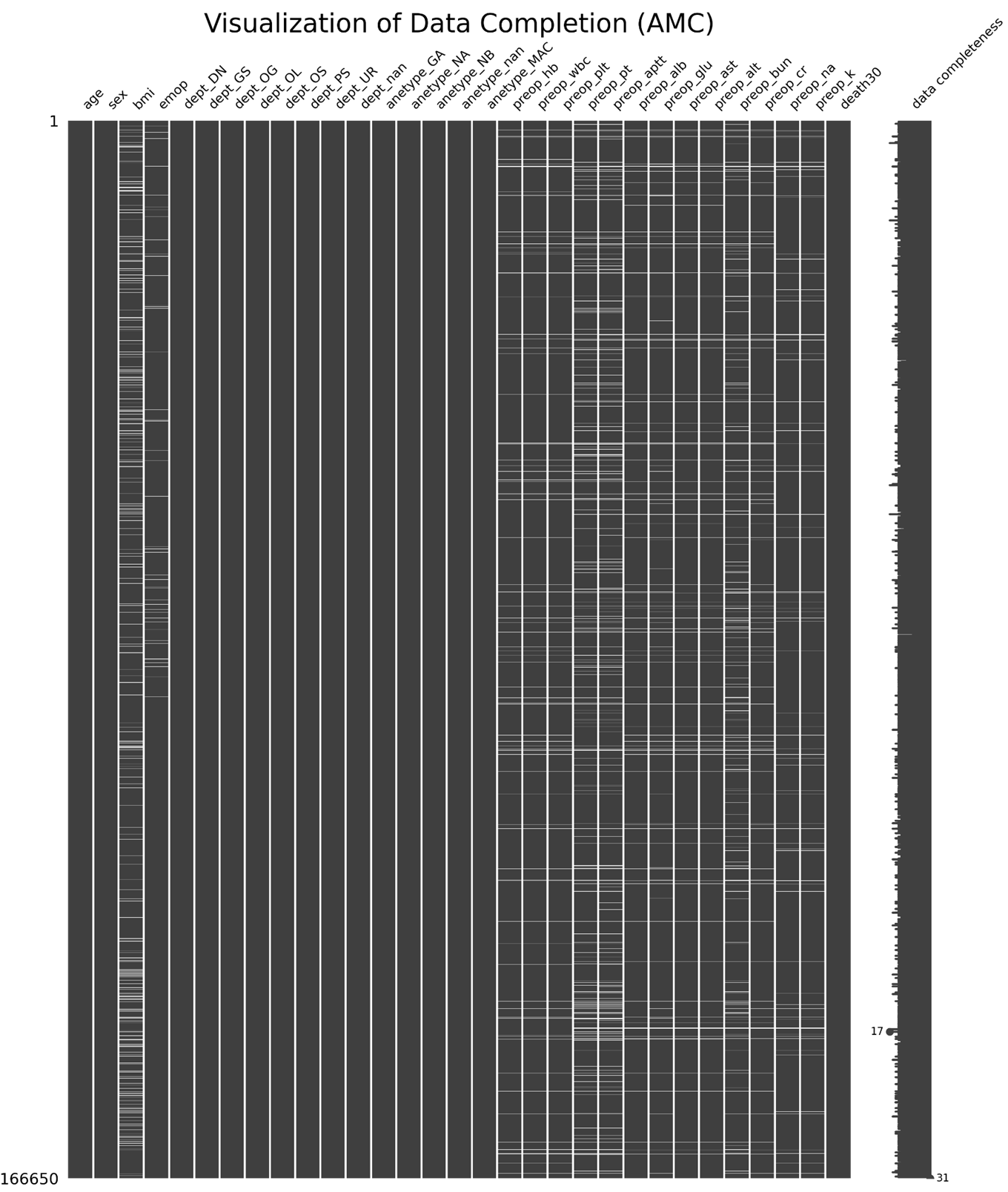

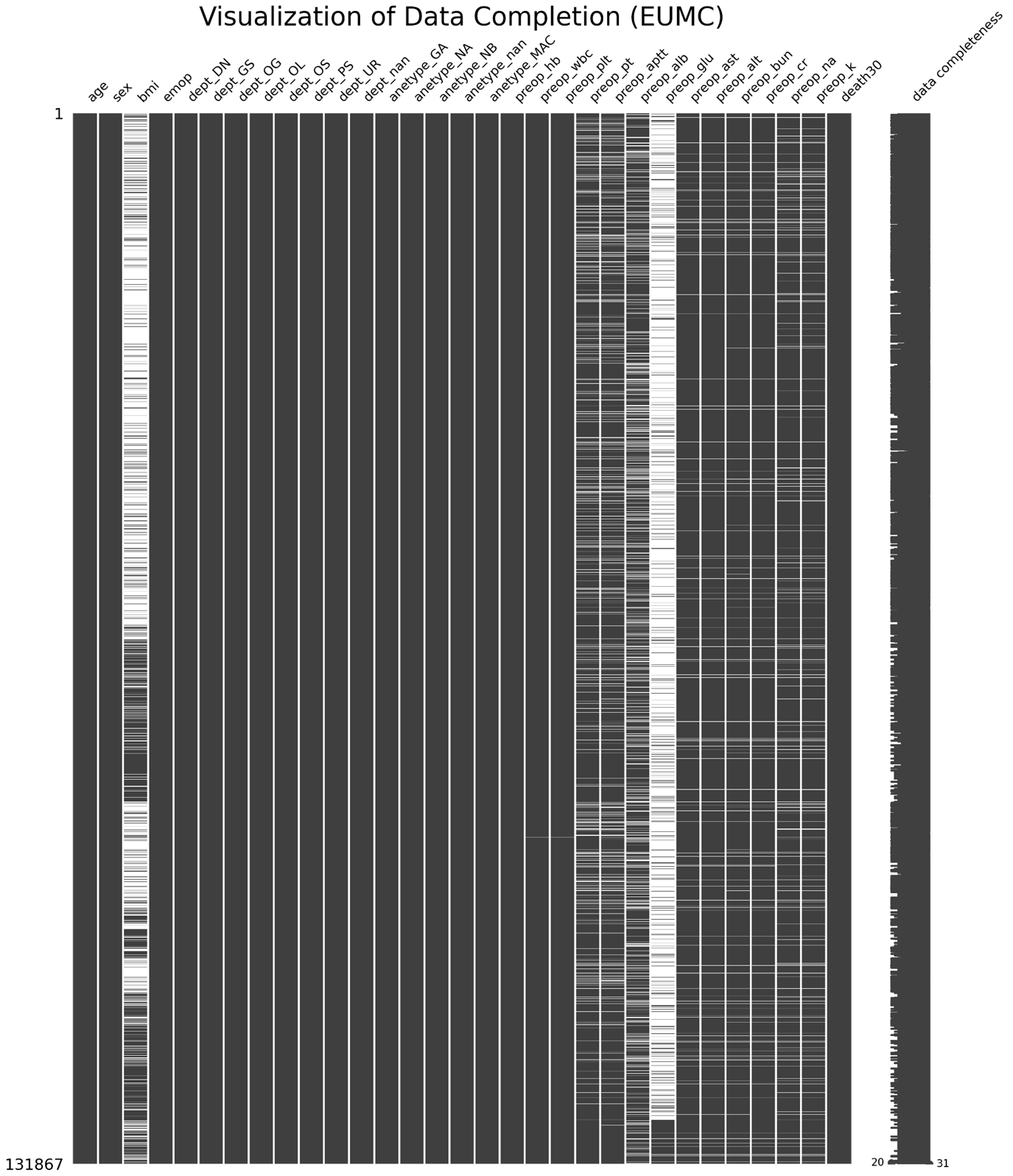


SNUH, Seoul National University Hospital; AMC, Asan Medical Center; EUMC, Ewha Womans University Medical Center.

**Figure S5. Nullity correlation heatmap in each hospital**


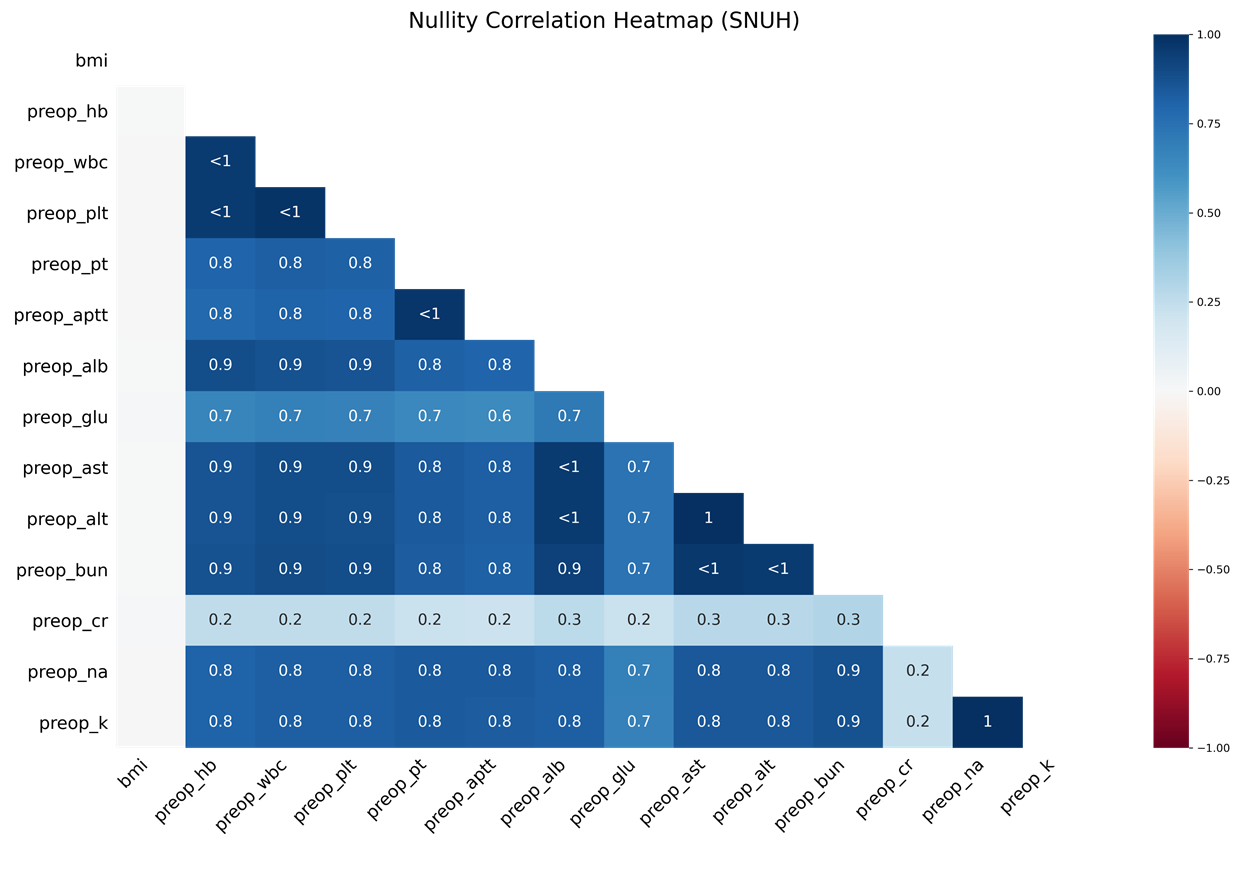

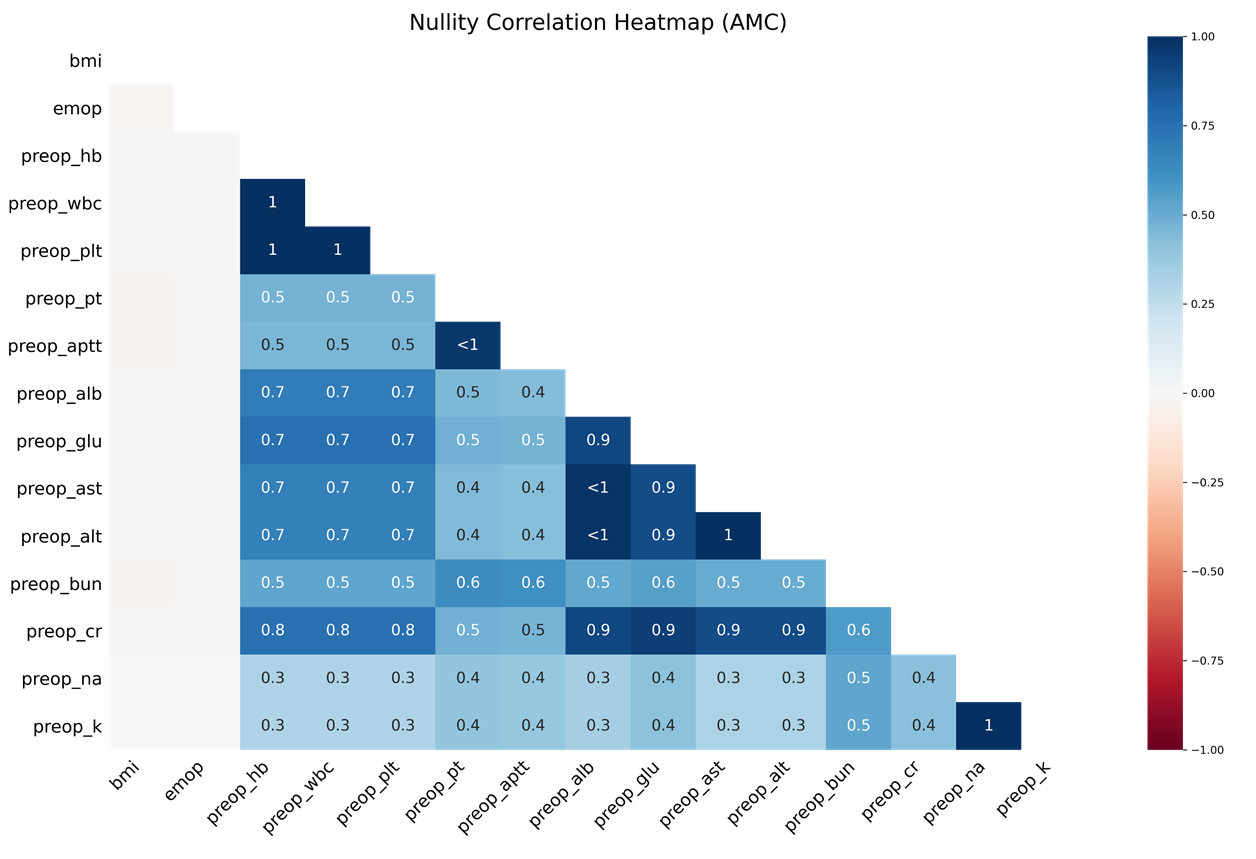


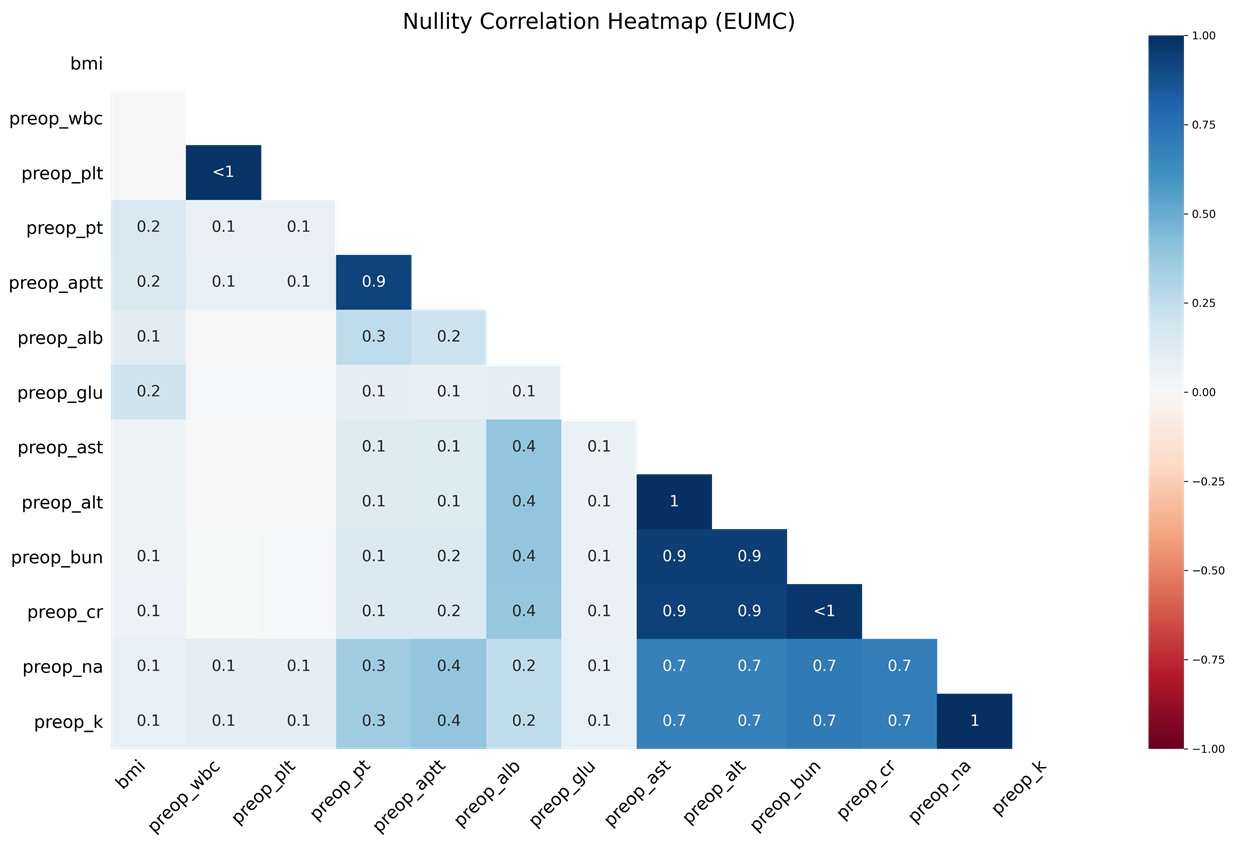


SNUH, Seoul National University Hospital; AMC, Asan Medical Center; EUMC, Ewha Womans University Medical Center.
